# Supplementation of Short-Chain Fatty Acid, Sodium Propionate, in Patients on Maintenance Hemodialysis: Beneficial Effects on Inflammatory Parameters and Gut-Derived Uremic Toxins, A Pilot Study (PLAN Study)

**DOI:** 10.3390/jcm7100315

**Published:** 2018-09-30

**Authors:** Stefania Marzocco, Gholamreza Fazeli, Lucia Di Micco, Giuseppina Autore, Simona Adesso, Fabrizio Dal Piaz, August Heidland, Biagio Di Iorio

**Affiliations:** 1 Department of Pharmacy, University of Salerno, 84084 Fisciano (SA), Italy; smarzocco@unisa.it (S.M.); autore@unisa.it (G.A.); sadesso@unisa.it (S.A.); 2 Rudolf Virchow Center, University of Wuerzburg, 97080 Wuerzburg, Germany; gh.fazeli@gmail.com; 3 UOC Nephrology, A. Landolfi Hospital, 83029 Solofra (AV), Italy; luciadimicco@gmail.com; 4 Department of Medicine and Surgery, University of Salerno, 84084 Fisciano (SA), Italy; fdalpiaz@unisa.it; 5 Department of Internal Medicine and KfH Kidney Center, University of Würzburg, KfH Kidney Center Würzburg, 97080 Würzburg, Germany; august.heidland@t-online.de

**Keywords:** propionic acid, chronic kidney disease, hemodialysis, gut microbiome, systemic micro-inflammation oxidative stress, indoxyl sulfate, *p*-cresyl sulfate

## Abstract

Background: In end-stage renal disease (ESRD), gut-derived uremic toxins play a crucial role in the systemic inflammation and oxidative stress promoting the excess morbidity and mortality. The biochemical derangement is in part a consequence of an insufficient generation of short-chain fatty acids (SCFA) due to the dysbiosis of the gut and an insufficient consumption of the fermentable complex carbohydrates. Aim of the study: The primary end-point was to evaluate the potential efficacy of SCFA (specifically, sodium propionate (SP)) for patients on maintenance hemodialysis (MHD) on systemic inflammation. Secondary end-points included potential attenuation of oxidative stress markers, insulin resistance and production of gut-derived uremic toxins indoxyl sulfate and p-cresol sulfate, as well as health status after SP supplementation. Study design: We performed a single-center non-randomized pilot study in 20 MHD patients. They received the food additive SP with a daily intake of 2 × 500 mg in the form of capsules for 12 weeks. Pre-dialysis blood samples were taken at the beginning, after six weeks and at the end of the administration period, as well as four weeks after withdrawal of the treatment. Results: The subjects revealed a significant decline of inflammatory parameters C-reactive protein (−46%), interleukin IL-2 (−27%) and IL-17 (−15%). The inflammatory parameters IL-6 and IFN-gamma showed a mild non-significant reduction and the anti-inflammatory cytokine IL-10 increased significantly (+71%). While the concentration of bacterial endotoxins and TNF-α remained unchanged, the gut-derived uremic toxins, indoxyl sulfate (−30%) and p-cresyl sulfate (−50%), revealed a significant decline. The SP supplementation reduced the parameters of oxidative stress malondialdehyde (−32%) and glutathione peroxidase activity (−28%). The serum insulin levels dropped by 30% and the HOMA-index by 32%. The reduction of inflammatory parameters was associated with a lowering of ferritin and a significant increase in transferrin saturation (TSAT). Four weeks after the end of the treatment phase, all improved parameters deteriorated again. Evaluation of the psycho-physical performance with the short form 36 (SF-36) questionnaire showed an enhancement in the self-reported physical functioning, general health, vitality and mental health. The SP supplementation was well tolerated and without important side effects. No patient had left the study due to intolerance to the medication. The SP supplementation in MHD patients reduced pro-inflammatory parameters and oxidative stress and improved insulin resistance and iron metabolism. Furthermore, SP effectively lowered the important gut-derived uremic toxins indoxyl and p-cresol sulfate. These improvements were associated with a better quality of life. Further controlled studies are required in a larger cohort to evaluate the clinical outcome.

## 1. Introduction

Advanced chronic kidney disease (CKD) and end-stage renal disease (ESRD) are associated with multiple comorbidities such as cardiovascular disease, anemia, mineral and bone disorders, malnutrition, body wasting, muscle loss (sarcopenia), neurological problems and infections resulting in poor survival. Important promoters of these complications are the systemic inflammation, enhanced production of reactive oxygen species (ROS), acquired immunodeficiency [1,2] and an impaired glucose and insulin homeostasis [3].

Systemic inflammation and oxidative stress in ESRD are induced by activation of the innate immune system involving monocytes, macrophages, granulocytes and cellular constituents (endothelial cell activation), as well as depletion of natural regulatory T cells, which impairs their ability to suppress inflammation [2,4]. The concomitant reduced humoral immunity is favored by presenting depletion of dendritic cells, an impaired phagocytic ability of monocytes and polymorphonuclear leukocytes (PMNs) [4].

The normal gut microbiota plays a prominent role in the maintenance of health and disease prevention. In various disease states such as obesity, type 2 diabetes, cardiovascular disturbances and auto-immune diseases, marked alterations in its composition and functions occurs [5,6]. Furthermore, in CKD patients and more pronounced in ESRD, gut microbiota is quantitatively and qualitatively different from healthy controls and contributes to uremic syndrome [7,8,9]. Due to concomitant disruption of the intestinal barrier function, noxious luminal products are translocated in the body’s internal milieu. The passage includes whole bacteria (going into mesenteric lymph nodes), endotoxins/lipopolysaccharide (LPS, cell wall components of the bacteria) and other noxious luminal products, which induce a persistent local (gut) and systemic inflammation [10,11]. The process is intensified by the generation of several uremic toxins such as indoxyl sulfate, *p*-cresyl sulfate and trimethylamine-N-oxide [12]. The accumulation of these compounds correlates with systemic inflammation, protein wasting and accelerated cardiovascular complications in hemodialysis patients [13]. The dysbiotic gut microbiome in CKD is associated with inflammation [14,15], immune dysregulation, insulin resistance (IR) [14,15,16], cardiovascular disease (CVD) and CKD progression [14,15,16,17].

The biochemical derangement in CKD and ESRD is in part a consequence of an insufficient generation of short-chained fatty acids (SCFA) due to the dysbiosis of the gut and a diet with low fiber content for prevention of hyperkalemia [18]. SCFA are produced in the distal small intestine and the colon by anaerobic bacteria following fermentation of complex carbohydrates [19]. They consist of 1–6 carbon atoms, and the three major compounds are acetic (two carbons), propionic (three carbons) or butyric acids (four carbons). SCFA contribute to the health of the gut (microbiome and mucosa) and the overall health of the host. They have been shown to exert anti-inflammatory, anti-oxidative, anti-diabetic, anti-cancer and antibacterial effects [20,21]. A dysbiotic gut microbiome and lower levels of SCFA contribute to various diseases such as inflammatory bowel disease [5,22,23], rheumatoid arthritis and multiple sclerosis [24,25]. 

Supplementation of SCFA exerts anti-inflammatory actions both in intestinal epithelial cells [26] and in the cardiovascular system [27]. They also positively influence autoimmune reactions [28,29]. In particular, SCFA enhances the formation of regulatory T cells in the colon, which are critical for regulating intestinal inflammation [30]. Furthermore, effector T cells such as Il-10-positive T cells are implicated [30]. Likewise, SCFA are involved in the control of body weight and insulin sensitivity [31], cholesterol synthesis [32] and retardation of progressive CKD [33].

The microbiota is markedly modulated by dietary factors. The unhealthy Western style diet with a high content of fat and carbohydrates negatively affects the gut microbiome. On the other hand, the amount and type of non-digestible carbohydrate influences its composition and function and favors the intestinal SFCA formation [34,35]. In CKD patients, a high dietary fiber intake resulted in decreased inflammation and all-cause mortality [36]. However, in ESRD, consumption of a fiber-rich diet is a risk factor for hyperkalemia, especially in oliguric and anuric dialysis patients. Therefore, they are advised to consume less vegetables and fruits [37,38]. In addition, the frequent antibiotic therapy [39] and iron supplementation modulate the microbiome [40]. Furthermore, the phosphate binder sevelamer was shown to reduce endotoxin levels and p-cresol sulfate [41].

Sodium propionate (SP) is one of the major compounds of SCFAs and is chemically composed of a carboxylic acid moiety and a small hydrocarbon chain with three carbon atoms, two oxygen atoms and the hydrogen atoms. In this study, we evaluated the effect of SP supplementation on inflammatory and metabolic markers, uremic toxins indoxyl sulfate and *p*-cresyl sulfate, as well as self-reported health status.

## 2. Subjects and Methods

This pilot study was performed at the “Ospedale “A Landolfi” (Solofra, Italy) and was conducted between August and October 2017. The study was accepted by the Ethical Committee of the Campania Nord, and it is registered at ClinicalTrials.gov (Number NCT02976688).

Study population: 20 stable MHD patients aged from 18–90 years were included (Table 1). We intentionally included elderly patients because the population of patients under renal replacement therapy becomes increasingly older and the microbiome is affected by aging, in part due to reduced renal function [42]. The average time on dialysis was 45 months. The most often occurring causes of renal failure were diabetes mellitus (*n* = 9) and chronic glomerulonephritis (*n* = 7). All subjects suffered from vascular complications. 

Written consent was obtained from all participants prior to the start of the project. Patients with severe malnutrition, infection, carcinoma, previous renal transplantation, intestinal diseases (medically-diagnosed irritable bowel syndrome, Crohn’s disease, ulcerative colitis and diarrhea) and antibiotic treatment within one month before and during the study were excluded. 

**Figure 1:**
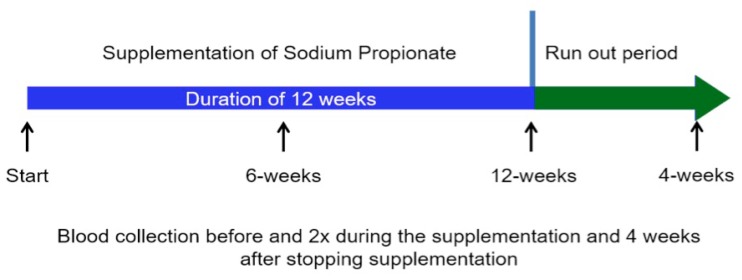
Representation of the study design. Blood samples were collected always before the start of the mid-week dialysis sessions.

Study design: All participants received the food additive sodium propionate (SP) with a daily intake of 2 × 500 mg in the form of capsules (Propicum^®^, Flexopharm Brain, Herne, Germany). Patients were asked not to change their eating habits to avoid a higher fiber intake during the experimental phase. The study design is illustrated in Figure 1.

### 2.1. Biochemical Measurements 

Serum inflammatory biomarkers: High sensitive C-reactive protein (hs-CRP), interleukin 2 (IL-2), IL-6, IL-10, IL-17a, tumor necrosis factor α (TNF-α), interferon (INFγ), transforming growth factor β (TGF-β) and endotoxin/lipopolysaccharide levels. 

IL-2, IL-6, IL-10, IFN-γ and TGF-β levels in sera were analyzed using an ELISA assay (#BMS213-2; #BMS215/2; #BMS221/2; #BMS228; #BMS249/4; respectively), according to the manufacturer’s instructions (e-Bioscience, San Diego, CA, USA). Serum TNF-α was measured with an enzyme-linked immuno sorbent assay (ELISA; #589201), according to the manufacturer’s instructions (Cayman Chemical, Ann Arbor, MI, USA). Sera were previously diluted in assay buffer (1:5), and then, the ELISA kit for TNF-α was performed. Bacterial endotoxin levels were evaluated by a limulus amebocyte lysate chromogenic endpoint assay (LAL; #HIT302), according to the manufacturer’s instructions (Hycult Biotech, Wayne, PA, USA). Sera were previously diluted in assay buffer (1:3), and then, the kit for LAL was performed. 

Serum oxidative stress biomarkers: malondialdehyde (MDA) and glutathione peroxidase (GPx) activity. The serum levels of MDA were analyzed using a commercial immunoassay kit (# E-EL-0060), according to the manufacturer’s instructions (Elabscience Biotechnology Co., Ltd., Houston, TX, USA). GSH-PX activity was evaluated using an ELISA kit (#e-BC-K096), according to the manufacturer’s instructions (e-Bioscience). 

Uremic toxins produced in the intestinal tract: *p*-cresyl sulfate and indoxyl sulfate. They were detected and quantified by an LC-MS/MS-based approach [43]. Plasma samples underwent a methanol-induced protein precipitation before the LC/MS/MS analyses. To that aim, 50 μL of each sample were added with 200 μL of methanol and mixed for 5 min. Samples were then centrifuged at 10,000× *g* at 4 °C for 15 min; the supernatant was collected and centrifuged again to obtain a clear extract. The supernatant was dried using a vacuum evaporator (Eppendorf, Hamburg, Germany), and the residue was dissolved in 50 μL of 10% methanol in 0.1% formic acid in water and centrifuged at 5000× *g* at 4 °C for 5 min to obtain a clear extract.

LC/MS/MS analyses were performed with an LC-MS/MS apparatus consisting of an Ultimate3000 UHPLC system (Thermo-Fisher, Waltham, MA, USA) coupled with a TSQ-endure mass spectrometer (Thermo-Fisher) equipped by an electrospray ion source and a triple quadrupole ion analyzer. Chromatography was performed using a Luna-Ω C18 column (50 × 2.1 mm; 1.6 μm) and a mobile phase composed of 0.1% formic acid (A) and acetonitrile (B). To achieve compounds elution, a gradient from 20% to 80% of B over 2 min was used; the total flow was 0.4 mL/min, and the injection volume was 2 μL. Mass spectra were acquired in negative selected reaction monitoring (SRM) mode: two transitions were selected for each compound in order to maximize selectivity and sensitivity. Specifically, the transitions 212 → 132 and 212 → 80 were chosen for indoxyl sulfate and 187 → 80 and 187 → 107 for *p*-cresyl sulfate. Direct injections of pure compounds were carried out in order to define suitable transitions and optimal instrumental parameters for each analyte. Compounds’ absolute quantization was achieved by fitting the peak area measured for each sample on external calibration curves. 

Glucose metabolism: Insulin resistance (IR) was determined with the HOMA Index (homeostasis model assessment) by measuring fasting blood glucose and insulin level, as well as hemoglobin HbA1c.

Iron metabolism: Iron, ferritin, transferrin and transferrin saturation (TSAT) were measured with the colorimetric method in human serum using ELISA.

Serum lipid levels: Triglycerides, total cholesterol, high and low-density cholesterol levels were measured using ELISA. 

Nutritional status: Serum albumin was measured using ELISA.

Parameters of well-being: patient-reported health using the short form 36 questionnaire (SF-36).

Treatment safety: SP is a non-toxic food additive and used as a conserving material in the baking industry. The European Food Safety Authority (EFSA) confirmed and licensed it: sodium propionate E281 [44]. SP is also allowed by the American Food and Drug Administration (FDA Select Committee on GRAS Substances (SCOGS)) since 1979 in amounts of up to 3000 mg/kg [45]. We performed an oral application of 500 mg SP 2× per day. This dose is about 0.014 mg/kg of the body weight. Therefore, no risk of toxicity is expected in the patients. 

### 2.2. Statistical Analysis 

To determine the effects of SP supplementation on biomarkers of inflammation and oxidative stress, we used 1-way repeated measures analysis of variance. These analyses were done using analysis of covariance. *p*-values < 0.05 were considered statistically significant. 

## 3. Results

The physical and general biochemical parameters are summarized in Table 2. Their average age was 70 years.

All of them performed a four-hour dialytic treatment three times a week using an arterio-venous fistula as vascular access. None of them changed the dialysis program during the study, and none had a central venous catheter. Biocompatible membranes were used for dialysis treatment (polysulfone (5), polymethylmethacrylate (5), polyamide (4), polyphenylene (6)). During the study, the previous kind of dialysis treatment was continued: 8 patients performed bicarbonate dialysis, 6 hemofiltration reinfusions (HFR) and 6 hemodiafiltrations online.

The SP supplementation was well tolerated, and no patient stopped the treatment program. Body weight remained stable. The systolic blood pressure decreased insignificantly by 10%, while the diastolic blood pressure remained unchanged. The blood concentrations of creatinine, urea, uric acid sodium, potassium, calcium, phosphate, bicarbonate, albumin and triglycerides remained unchanged, while LDL-cholesterol showed a non-significant decrease (Table 2).

Blood glucose and HbA1c remained unchanged. The insulin values dropped by 30% and the HOMA-index by 32%. There was no difference in the improved glucose metabolism between diabetic and non-diabetic patients in our small group of investigated patients. Hemoglobin showed a trend to higher levels, associated with a better iron utilization (increase of TSAT) and a reduction of ferritin levels (Table 2).

Determination of the inflammatory parameters showed a significant decline of hs-C-reactive protein (−46%), IL-2 (−27%), IL-17 (−15%) and TGF-β (−20%). The anti-inflammatory cytokine IL-10 increased (+71%) significantly (Table 3). IL-6 and IFN-γ revealed a mild non-significant reduction. The concentrations of endotoxins and TNF-α remained unchanged (Table 3). MDA levels were significantly decreased (−32%), and GSH-PX activity dropped by 28% (Table 3). Importantly, indoxyl sulfate (−30%) and *p*-cresyl sulfate (−50%) were significantly reduced during SP treatment (Table 3). Four weeks after withdrawal of SP supplementation, all improved parameters deteriorated again. 

These positive biochemical changes corresponded to an improvement of the self-reported psycho-physical performances after 12 weeks, as evidenced by the score of SF-36 questionnaire (Table 4). After withdrawal of SP supplementation, no additional SF-36 assessment was performed.

No significant side effects (neither intradialytic, nor during the interdialytic period) were recorded during the use of SP, and no patient had left the study due to intolerance to SP.

## 4. Discussion 

To the best of our knowledge, the effect of SP on the patho-biochemistry of patients with ESRD undergoing MHD has not been investigated. This is the first report about the beneficial impact of SP in this patient group. We observed striking improvements of inflammatory and metabolic biomarkers, as well as self-reported wellbeing. SP supplementation resulted in a significant decrease of hs-CRP, IL-2 and IL-17a, as well as an insignificant decline of IL-6 and IFN-γ. Interestingly, also the circulating levels of TGF-β were ameliorated. This cytokine has numerous functions and is an important pathogenetic factor of the fibrosis of various organs and the cardiovascular system in ESRD [46]. The anti-inflammatory cytokine IL-10 showed a significant rise. 

Further, parameters of oxidative stress (malondialdehyde and glutathione peroxidase) were improved. Reactive oxygen species (ROS) are constantly formed inside cells and play a pivotal role in signaling and defense against microbial pathogens [47]. The source of oxygen radicals is mainly mitochondrial oxidative phosphorylation, as well as oxidant enzymes, including myeloperoxidase. ESRD is usually associated with higher levels of oxygen radicals, which can damage proteins, DNA, lipids and other macromolecules in the body [48]. We observed that SP supplementation ameliorated MDA concentration and glutathione peroxidase activity (Table 3). We did not measure the concentration and activity of myeloperoxidase as an important source of reactive oxygen species in kidney diseases and numerous other chronic inflammatory disturbances [49,50]. However, our data suggest that the overall concentrations of oxygen radicals were reduced. Since systemic inflammation and oxidative stress are regarded as key mediators of the enhanced cardiovascular morbidity and mortality in ESRD [51], their long-term amelioration could promote the health status of the patients with an increased life expectation, although this has not been experimentally shown.

The improved inflammation and oxidative stress were associated with an amelioration of the impaired glucose metabolism. SCFA, in particular propionic acid, has been shown to ameliorate the glucose homeostasis and insulin resistance [31,52]. This may be caused by improved beta cell function with stimulation of insulin secretion and an increase in glucagon-like peptide-1 secretion, which participates in glucose homeostasis by lowering plasma glucose concentration and improving insulin resistance [53]. We found after SP supplementation a decline of fasting insulin level and a betterment of the HOMA-index. Surprisingly, fasting glucose and HbA1c levels remained unchanged. The value of HbA1c as a parameter of long-time plasma glucose concentration in ESRD is limited because of the shortened red cell survival in these patients. An unchanged HbA1c level despite an improved insulin resistance can be theoretically related to the longer life of erythrocytes due to the anti-inflammatory effects of SP. In fact, we observed a small rise in hemoglobin levels. In ESRD patients, increased insulin resistance accelerates muscle protein degradation [54] and the development of atherosclerosis and cardiovascular mortality [55]. Therefore, an improved glucose homeostasis may delay these complications.

Since inflammation is also known to influence iron status, we examined various parameters of iron metabolism after SP supplementation. We found a decline of ferritin concentration and a significant increase in the transferrin saturation. These alterations are most probably induced due to the improved systemic inflammation [56,57]. In addition to being a marker of iron stores, ferritin also increases with inflammation and is further related to mortality [56,57]. Moreover, inflammation contributes to erythropoietin resistance [58], which could be improved after long-term SP supplementation, in line with our observed trend to higher hemoglobin levels. 

The systolic blood pressure showed an insignificant decrease of 10%, while the diastolic pressure was unchanged. There is growing evidence that the SCFA are involved in blood pressure modulation control [59,60]. However, the mechanisms of these interactions are not fully understood. Recent in vitro and animal studies evidence shows that blood pressure is affected by interacting with host GPCRs, including GPR41 and Olfr78. Indeed, mice null for Olfr78 are hypotensive, and mice null for Gpr41 are hypertensive, highlighting mechanisms that physiologically intervene on SCFAs and blood pressure control [59]. 

The improvement of the inflammation and oxidative stress could be in part a consequence of the lowering of key mediators of gut-derived toxins. A multitude of animal and clinical studies showed that indoxyl and *p*-cresyl sulfate contribute to cardiovascular disease and an enhanced mortality in ESRD [61,62]. Serum levels of indoxyl sulfate were significantly associated with aortic calcification and pulse wave velocity [63]. Both serum indoxyl sulfate and *p*-cresyl sulfate correlate with systemic inflammation and mortality rate [64,65]. Although the intestinal microbiome has not been evaluated, the significant lowering of the indoxyl and *p*-cresyl sulfate concentration could be a consequence of a change in the intestinal pathobionts by a decrease in relative abundance of bacteria possessing p-cresol and indole-producing enzymes to produce indoxyl sulfate and *p*-cresyl sulfate with a potential increase of *Bifidobacterium* spp. [18,64,65]. However, the blood levels of endotoxins remained unchanged. This finding suggests that the pathobionts involved in the enhanced bacterial endotoxin abundance were not influenced by the SP within the 12-week treatment period. 

The beneficial actions of SCFA have been attributed to the following pathways: the G-protein-coupled receptors GPR 41 and GPR 43 (the free fatty acid receptors FFAR3, FFAR2) and Olfr78, the inhibition of histone deacetylases (HDAC) and stimulation of histone acetyltransferase (HAT) activity [59]. GPR 43 has a potential role in inflammation and metabolic receptors. These receptors are found in human colonic tissue, in white adipose tissue, skeletal muscle and liver [66]. In the distal gut, SCFA bind to GPR41 and GPR43 leading to the production of PYY and GLP-1, which affects glucose homeostasis and lipid accumulation [53]. 

Propionic acid is involved in most effects of the short chain fatty acids including inhibition of intestinal and hepatocyte lipid synthesis [67], lowering of fasting glycemia [68] and protection against diet-induced obesity [69]. SP also regulates colonic T-reg cell homeostasis [29,30] and exerts marked anti-inflammatory actions including intestinal epithelial cells and macrophages [70], as well as in neutrophils, colon cells and colon cultures [71,72]. It improved experimental autoimmune encephalomyelitis [6] and experimental acute renal failure [73]. In addition, antibacterial effects were also documented [74,75]. 

Various strategies have been proposed to attenuate the microbial alterations in ESRD [14]. They include: use of prebiotics, probiotics and their combination. Prebiotics are especially carbohydrates that are nearly or wholly indigestible and that, when consumed in food, promote the growth and activity of beneficial bacteria in the digestive tract. Prebiotic therapy with complex carbohydrates was successfully administered in hemodialysis patients [76]. Recently, the effect of high amylose-resistant starch supplementation was shown to improve parameters of inflammation and oxidative stress in animal experiments [77], as well as in hemodialysis patients [76]. In the latter study, the C-reactive protein concentration and total antioxidant activity was not changed, but, serum urea and creatinine concentration significantly declined. Probiotic therapy is the administration of live microbial species to prevent the growth of harmful bacteria and restore the normal intestinal flora. Use of probiotics showed mixed results. Some authors found it to be ineffective in the modulation of inflammation in ESRD [10]. A combination of prebiotics and probiotics, the so-called synbiotic therapy, resulted in a significant lowering of *p*-cresyl sulfate, but not indoxyl sulfate. Furthermore, activated charcoal (AST-120) has been used to attenuate the gut microbial alterations and the systemic inflammation. In animal studies and small patient groups, the compound lowered inflammatory markers and the plasma levels of indoxyl sulfate and *p*-cresyl sulfate and retarded the progression of CKD [78,79]. However, in a randomized controlled study, AST-120 did not slow the CKD progression [80].

Our investigation suffers from a few limitations: (1) small number of patients; (2) short duration of the study; (3) being a monocentric study; (4) nutrition intake was not recorded; and (5) no analysis of gut bacteria.

## 5. Conclusions

In conclusion, our pilot study provided new insights about the benefits of SP on systemic inflammation, oxidative stress, metabolic disturbances (in particular in insulin resistance and iron metabolism) and the formation of the key intestinal uremic toxins, indoxyl sulfate and *p*-cresyl sulfate. Further, the patient-reported quality of life was improved. Adequately powered, randomized-controlled trials are needed to assess the effects of propionate supplementation in MHD patients.

## Figures and Tables

**Table 1:** Characteristics of the 20 hemodialysis patients.

**Parameter**	**Mean ± SD**
Age (years)	69.9 ± 11.2
Gender (male/female)	11/9
Height (cm)	166 ± 6
Weight (kg)	64.8 ± 13.5
BMI (kg/m^2^)	26.1 ± 2.5
Dialysis vintage (months)	45 ± 19
**Causes of renal failure**	***n*** **(%)**
Chronic glomerulonephritis	7 (35)
Polycystic kidney disease	2 (10)
Chronic pyelonephritis	2 (10)
Diabetes mellitus	9 (45)
**Comorbidities**	***n*** **(%)**
Hypertension	18 (90)
Cerebral vasculopathy	4 (20)
Peripheral vasculopathy	11 (55)
Recurrent cystitis	1 (5)
Chronic obstructive pulmonary disease	2 (10)

**Table 2:** Physical and biochemical parameters at the study baseline, after 6 and 12 weeks of intervention and 4 weeks after withdrawal of the sodium propionate (SP) supplementation.

	Start	6 Weeks	12 Weeks	16 Weeks	*p*
Body weight (kg)	64.8 ± 13.5	64.2 ± 13.5	64.3 ± 13.0	64.5 ± 13.2	NS
Systolic blood pressure (mm Hg)	144 ± 39	135 ± 41	130 ± 40	140 ± 49	NS
Diastolic blood pressure (mm Hg)	70 ± 11	67 ± 11	69 ± 12	72 ± 13	NS
Creatinine (mg/dL)	7.6 ± 2.5	7.7 ± 2.6	7.7 ± 2.9	7.8 ± 2.0	NS
Urea (mg/dL)	131 ± 42	129 ± 45	127 ± 55	133 ± 52	NS
Uric acid (mg/dL)	5.2 ± 1.4	5.4 ± 1.6	5.5 ± 1.7	5.2 ± 1.9	NS
Na (mmol/L)	137 ± 3	138 ± 3	139 ± 5	139 ± 6	NS
K (mmol/L)	5.4 ± 0.9	5.1 ± 0.8	5.0 ± 1.1	5.2 ± 1.0	NS
Ca (mg/dL)	8.9 ± 1.0	9.0 ± 0.7	9.1 ± 0.8	9.0 ± 0.9	NS
Phosphate (mg/dL)	4.4 ± 1.5	4.4 ± 2.0	4.2 ± 1.9	4.3 ± 2.1	NS
Alkaline phosphatase (U/L)	120 ± 69	134 ± 74	130 ± 70	142 ± 81	NS
PTH intact (ng/L)	259 ± 148	330 ± 152	301 ± 132	288 ± 178	NS
Bicarbonate (mmol/L)	22.6 ± 2.6	23.0 ± 2.7	23.0 ± 2.2	23.0 ± 2.5	NS
Albumin (g/dL)	3.6 ± 0.4	3.8 ± 0.3	3.8 ± 0.4	3.7 ± 0.5	NS
LDL-cholesterol (mg/dL)	116 ± 21	110 ± 24	102 ± 42	110 ± 31	NS
Triglycerides (mg/dL)	191 ± 103	195 ± 98	189 ± 102	199 ± 123	NS
Hemoglobin (g/dL)	11.6 ± 1.4	12.2 ± 1.5	12.3 ± 1.4	12.0 ± 1.8	NS
Serum iron (µg/dL)	51 ± 27	55 ± 22	55 ± 22	48 ± 33	NS
Transferrin (g/L)	180 ± 44	204 ± 53	221 ± 73	170 ± 49	NS
TSAT (%)	22 ± 15	39 ± 18	40 ± 20	25 ± 19	0.002
Ferritin (ng/mL)	226 ± 258	63 ± 80	73 ± 77	212 ± 188	0.01
Glucose (mg/dL)	124 ± 18	121 ± 16	120 ± 19	125 ± 20	NS
HbA1 c (%)	6.6 ± 1.2	6.7 ± 1.2	6.6 ± 1.3	6.7 ± 1.4	NS
Insulin (IU/mL)	27 ± 26	19 ± 15	20 ± 15	29 ± 28	0.024
HOMA-Index	11.7 ± 13.8	8.3 ± 7.4	8.1 ± 7.0	12.1 ± 9.6	0.041
Kt/V	1.31 ± 0.6	1.29 ± 0.3	1.32 ± 0.4	1.30 ± 0.5	NS
nPCR	1.10 ± 0.15	1.16 ± 0.29	1.18 ± 0.32	1.11 ± 0.19	NS

PTH: parathormone, TSAT: transferrin saturation, Kt/V: parameter of dialysis efficacy; nPCR: normalized protein catabolic rate.

**Table 3:** Markers of inflammation, oxidative stress and gut-derived toxins at study baseline, 6 and 12 weeks after the intervention and 4 weeks after stopping SP supplementation.

	Start	6 Weeks	12 Weeks	16 Weeks	*p*
hs-C-reactive Protein (mg/L)	5.98 ± 3.7	3.6 ± 6.3	3.2 ± 4.3	6.0 ± 4.2	0.003
IL-2 (pg/mL)	7.79 ± 2.3	6.16 ± 2.22	5.69 ± 2.02	7.95 ± 3.1	<0.007
IL-6 (pg/mL)	2.06 ± 1.36	1.79 ± 0.94	1.72 ± 0.84	2.1 ± 1.5	NS
IL-10 (pg/mL)	1.57 ± 0.62	2.59 ± 1.32	2.69 ± 1.23	1.47 ± 0.81	<0.001
IL-17 (pg/mL)	240 ± 37	212 ± 24	204 ± 17	244 ± 44	<0.01
TNF-α (pg/mL)	38.8 ± 12.6	38.5 ± 13.5	37.4 ± 8.3	38.4 ± 12.2	NS
IFN-γ (pg/mL)	0.49 ± 0.47	0.39 ± 0.33	0.36 ± 0.18	0.52 ± 0.45	NS
Endotoxin (EU/mL)	1.99 ± 0.88	2.36 ± 0.97	2.3 ± 0.79	2.02 ± 1.02	NS
TGF-β (pg/mL)	620.5 ± 220.9	505.1 ± 147.8	495.0 ± 152.7	612.4 ± 214.2	<0.05
Malondialdehyde (ng/mL)	389.2 ± 133.6	287.8 ± 103	266.8 ± 92.2	368.9 ± 109	<0.01
GSH-PX activity (micromole/L)	127.2 ± 75.3	94.5 ± 53.5	92.4 ± 52.4	138.4 ± 102.2	<0.01
Indoxyl sulfate (mg/L)	73.1 ± 29.3	58.9 ± 23.3	50.9 ± 24.0	70.7 ± 26.7	<0.001
P-cresol sulfate (mg/L)	37.9 ± 27.3	24.9 ± 13.8	19.1 ± 12.6	35.5 ± 24.7	<0.001

GSH-PX activity: glutathione peroxidase activity; parameter of oxidative stress.

**Table 4:** Evaluation of the quality of life assessed by the short form 36 (SF-36) questionnaire.

	Before SP Supplementation	After SP Supplementation	*p*-Value
Physical Components	58 ± 21	80 ± 19	0.001
Physical Functions	25 ± 15	33 ± 18	0.148
Pain Tolerance	35 ± 16	58 ± 17	0.001
General Health	27 ± 20	34 ± 16	0.152
Vitality	23 ± 10	45 ± 18	0.001
Social Functions	41 ± 16	70 ± 14	0.001
Role Emotional	33 ± 17	33 ± 13	0.948
Mental Health	29 ± 18	48 ± 22	0.005
